# Dying to Survive—The p53 Paradox

**DOI:** 10.3390/cancers13133257

**Published:** 2021-06-29

**Authors:** Andrea Lees, Tamas Sessler, Simon McDade

**Affiliations:** Patrick G Johnston Centre for Cancer Research, Queen’s University, Belfast BT9 7AE, UK; t.sessler@qub.ac.uk

**Keywords:** p53, cell death, apoptosis, targeted therapy

## Abstract

**Simple Summary:**

p53 is best known for its tumour suppressive functions mediated through regulation of an extensive-gene regulatory network. Here we review progress in our understanding of the complex role that p53 plays in controlling expression of cell-death regulatory pathways; how paradoxically, this may be important for cellular and organismal survival in response to homeostatic stress; the potential impact on the response to p53 activating therapies; and the ways that this might be exploited in cancer types for which maintaining wild-type p53 is beneficial.

**Abstract:**

The p53 tumour suppressor is best known for its canonical role as “guardian of the genome”, activating cell cycle arrest and DNA repair in response to DNA damage which, if irreparable or sustained, triggers activation of cell death. However, despite an enormous amount of work identifying the breadth of the gene regulatory networks activated directly and indirectly in response to p53 activation, how p53 activation results in different cell fates in response to different stress signals in homeostasis and in response to p53 activating anti-cancer treatments remains relatively poorly understood. This is likely due to the complex interaction between cell death mechanisms in which p53 has been activated, their neighbouring stressed or unstressed cells and the local stromal and immune microenvironment in which they reside. In this review, we evaluate our understanding of the burgeoning number of cell death pathways affected by p53 activation and how these may paradoxically suppress cell death to ensure tissue integrity and organismal survival. We also discuss how these functions may be advantageous to tumours that maintain wild-type p53, the understanding of which may provide novel opportunity to enhance treatment efficacy.

## 1. Introduction

In order to survive, all multi-cellular organisms must undergo cell death. This process occurs during both embryonic development and in the mature adult when cells that become obsolete, irreversibly damaged or potentially harmful to the organism are eliminated in an active and controlled manner. The dedicated molecular machinery responsible for executing such physiological or ‘programmed’ cell death pathways must therefore be tightly regulated to maintain homeostasis and avoid aberrant initiation of terminal cell fates. Indeed, dysregulation of cell death has been linked to the pathogenesis of various diseases such as cancer, neurodegenerative disorders and autoimmune and inflammatory conditions [1]. Therapeutically exploiting aberrations in the initiation and propagation of cell death is therefore of immense clinical importance, hence research in this area has been extensive although not yet exhaustive as new, regulated forms of cell death continue to be identified and explored. Some 12 distinct cell death programs have now been described which include canonical apoptosis as well as emerging pathways such as necroptosis, ferroptosis, and pyroptosis [2]. Whilst diverse with respect to their activatory stimuli and the core molecular components that disseminate and execute the death signal, all of these pathways aim to achieve the same end-goal—the regulated destruction and removal/recycling of the cell. Importantly, it is increasingly clear that differences in the ‘choice’ of cell death mechanisms, has wider implications than just the individual cell itself, since signaling to the local microenvironment is often necessary to prevent the spread of damage and restore overall homeostasis [3]. For example, the release of damage-associated molecular patterns (DAMPs) from cells succumbing to infection or undergoing serious stress alerts and alters signaling in nearby cells to facilitate clearance and preserve the function of the surrounding tissue [4]. Even the historically immune inert, non-inflammatory process of apoptotic cell death has recently been shown to result in actively secreted metabolites that modulate specific gene programs in neighbouring cells [5]. Thus, committing a cell to death should be a carefully considered, deliberate act which balances both the consequences for an individual cell or the organism as a whole.

This is particularly evident in the context of oncogenesis wherein resistance to cell death is recognised as a hallmark event in cancer initiation and progression [6,7]. Failure to eliminate cells which have undergone neoplastic transformation results in the expansion and survival of malignant cell populations which, without intervention, is often to the detriment of the entire organism. Thus, survival mechanisms must be put in place which through the activation of programmed cell death, paradoxically ensure survival. In this respect, the transcription factor and prominent tumour suppressor p53 is often hailed as a master regulator of cell fate. In the 40 years since its discovery, intensive studies of the p53 response and the plethora of genes it regulates has been shown to elicit effects across many of the most fundamental cellular pathways and regulatory networks that govern homeostasis throughout the body [8,9,10,11]. However, in its best described role as a “guardian of the genome”, p53 plays a central role in regulating the response to DNA-damage by balancing the activation of cell-cycle arrest and DNA-damage repair to promote cell survival versus activating apoptosis and cell death [12]. This ensures that potentially harmful DNA aberrations are not potentiated through cellular replication which ultimately suppresses tumour development. So vital is this role that inactivation or suppression of certain functions of p53 is thought to be a prerequisite in most, if not all, human cancers. Extensive mapping of tumour DNA, facilitated by the explosion of routine next generation sequencing (NGS) techniques, has revealed that around 50% of all tumours harbour a mutation in the *TP53* gene (which encodes the p53 protein), making it the most frequently mutated gene in human cancer [13]. Mutational inactivation of the wild-type tumour suppressive function of p53 is highly effective, however it is not the only means by which p53 activity is abrogated. Tumour cells retaining wild type p53 expression employ myriad mechanisms to disrupt p53 signalling upstream and/or downstream of its activation, many of which are now targetable [14,15]. Reactivating the tumour suppressive functions of p53, particularly those in favour of cell death, is therefore a highly attractive therapeutic strategy which has already shown clinical promise. It is therefore perhaps surprising that tumours retaining wild-type p53 in many cases do not necessarily have better outcomes in response to treatment modalities such as chemo- and radiotherapy. This review aims to discuss the role of activated p53 in mediating cell death, strategies which cancer cells employ to deregulate this process and the therapeutic potential and challenges of harnessing p53 activation to promote cell death and tumour regression.

## 2. p53—A Tumour Suppressive Transcription Factor

**Figure 1:**
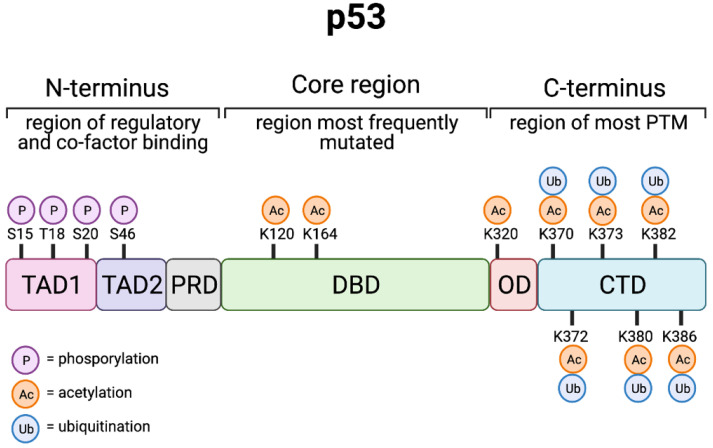
p53 structure and post-translational modification. Schematic representation of distinct p53 domains and the key post- translational modifications which have been shown to regulate p53 activity; transactivation domain (TAD1 and TAD2), proline-rich domain (PRD), DNA-binding domain (DBD), oligomerisation (OD), and the C-terminal domain (CTD). Created with BioRender.com (accessed on 15 May 2021).

The tumour suppressor p53 functions as a sequence-specific transcription factor which binds to the regulatory regions of target genes in order to induce their expression and control cell fate [11,16,17]. Canonical “wild-type” p53 (also denoted TAp53α) is the most extensively studied p53 isoform of which 9 have been reported [18]. Encoded by the *TP53* gene, wild-type p53 is expressed as a 393 amino acid peptide composed of several distinct domains (Figure 1). Two tandem transactivation domains (TAD1 and TAD2) are present at the p53 N-terminus which bind both positive (p300/CBP) and negative (MDM2/MDMX) regulators of its transcriptional activity and protein stability [19]. Such proteins typically enact their regulatory effects through the addition or removal of post-translational modifications, an abundance of which are found within the lysine-rich C-terminal domain. The immediately adjacent proline rich domain (PRD) has been implicated in regulating the apoptotic function of p53 [20]. The main core of the p53 protein contains the DNA-binding domain (DBD), which consistent with its function as a tumour suppressive transcription factor, is the site most frequently mutated in cancer [21,22]. In order to efficiently bind to DNA at specific target sites, p53 must first form a homo-tetrameric structure which is facilitated by the oligomerisation domain (OD) and to some extent the C-terminal domain (CTD). The putative DNA binding motif responsible for the recruitment of p53 to particular target genes is known as a p53 response element (p53RE) and is comprised of two decameric half sites of the consensus sequence RRRCWWGYYY (R = G/A; W = A/T; Y = C/T) separated by a spacer of between 0 and 13 base pairs [23,24]. Typically, a gene-proximal p53RE located within 2.5 kb of the transcriptional start site is necessary for the transactivation of so-called ‘direct’ p53 targets, although binding also frequently occurs within the first intron. How p53REs enable the discrimination and activation of specific genomic targets to induce particular p53-driven cell fates has been an area of intense research. Indeed, numerous computational, biochemical and multi-omic approaches have been employed in efforts to more robustly examine the sequence and context specificity of p53REs and correlate direct binding with gene expression and cell fate [25,26,27,28,29].

An emerging concept is that high affinity p53REs are well conserved across species and are present at cell cycle arrest genes (e.g., *CDKN1A*/p21) whilst lower affinity, evolutionary divergent p53REs are found at pro-apoptotic genes (e.g., *BAX*/BAX) [30,31]. However, the underlying molecular mechanisms which dictate p53 binding at distinct p53REs remain poorly defined. Conserved G/C residues at positions 4, 7, 14, and 17 of the p53RE mediate tetrameric p53 binding at specific genomic targets with mismatches in the core ‘CWWG’ typically resulting in lower binding affinity [32,33]. Interestingly, such mismatches have been reported in both high- and low-affinity targets and indeed the number of mismatches within a specific p53RE is not simply predictive of differential affinity. In a recently described model, alterations in the p53RE nucleotide code at positions 3, 8, 13, and 18 create a specific DNA shape that is sensed by two p53 DNA contact residues (Lys120 and Arg248) resulting in differential p53 interactions and a change in conformation [34,35]. This facilitates p53 binding to specific high- and low-affinity p53REs which modulates target gene expression and cell fate when manipulated in vivo [35]. However, in addition to sequence specificity, the functional state of the DNA, chromatin and p53 itself (in terms of post-translational modification) and indeed the cell type and mechanism of p53 activation also play important roles in determining downstream target activation and subsequently cell fate [36,37,38,39]. Importantly, efforts to comprehensively map p53REs may be encumbered by their frequent occurrence in respective, often methylated regions (which can be hard to map genomically with short read technologies) of the genome driven by proliferation of LINE elements [40]. The emerging importance of p53 and such elements in repressing retrotransposons during development and the potential vulnerability this imposes in p53 deficient cancers requires further study of the non-canonical role of p53 binding to these “dark” regions of the genome [41,42,43].

Interestingly, distal p53 binding sites have also been identified in regions such as enhancers which adds a growing degree of complexity when trying to correlate p53 binding with direct gene expression (reviewed in [11]). Moreover, whether p53 can function as both a direct activator and repressor of transcription has been an area of intense study and debate. Recent large-scale meta-analyses of published ChIP-seq and gene expression data have aimed to reconcile this paradigm [26,27,44], with a generally emerging concept that p53 functions as a direct transcriptional activator with resulting target gene expression acting to indirectly repress transcription in a p53-dependent manner [45]. Thus, p53 activation can result in the direct and indirect regulation of multiple signalling pathways which regulate almost all aspects of cell fate. This includes canonical activation of cell-cycle arrest, DNA-repair, senescence, and apoptosis as well as emerging roles in autophagy, metabolism and the immune response.

These meta-analyses identify a core of ~500 protein-coding genes as being directly regulated by p53-mediated transcriptional promoter activation; although exploration of how p53 affects a larger spectrum of genes in a tissue specific manner through interactions with enhancers and non-functional regions of the genome through pioneer factor activity is an area of intensive investigation [11]. Direct transactivation leads to the upregulation of well-established p53 targets such as *CDKN1A* (p21), *MDM2* (MDM2), and *BBC3* (PUMA), the functions of which have been extensively mapped and studied (reviewed in [46]). However, as our understanding of novel protein interactions and cellular pathways increases, the roles of even the most renowned p53 targets, as well as those yet to be fully explored, continue to be expanded and defined. Thus, the transcriptional activation of p53 targets likely spans a multitude of both distinct and overlapping cellular pathways which are regulated in a context dependent manner (i.e., cell type and stimuli). Indeed, in terms of its ability to induce cell death, p53 is canonically recognised as a master regulator of apoptosis. However, with the discovery and characterisation of novel forms of regulated cell death, diverse roles for established p53 induced targets as well as new p53 transcriptional targets continue to be identified.

Nonetheless, the canonical tumour suppressive functions of p53 are still heavily intertwined with its long-established role in activating apoptotic cell death. Whilst this involves transcriptional upregulation of direct activators of apoptosis—such as PUMA, NOXA, and BAX—numerous studies have also described transcription-independent roles for p53 in facilitating apoptotic cell death. However, simply activating p53 does not immediately condemn a cell to apoptotic cell death as a host of regulatory proteins are simultaneously regulated in order to prevent unnecessary commitment to this terminal and, largely, irreversible fate.

## 3. Apoptosis and Beyond

**Figure 2:**
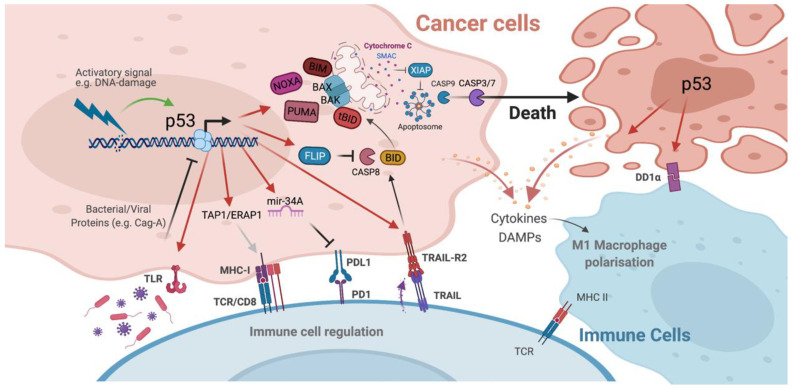
p53 regulates cell death and immune responses. Schematic representation of the core proteins and signalling molecules involved in p53-induced cell death and immune cell regulation. Created with BioRender.com (accessed on 12 June 2021).

In mammalian cells, apoptosis is induced by two distinct, yet ultimately converging signalling pathways known as the extrinsic (death-receptor-mediated) and intrinsic (mitochondrial-mediated) apoptotic pathways (Figure 2) [47]. Activation of intrinsic apoptosis occurs in response to a diverse range of intracellular stimuli including oncogenic stress, DNA-damage, nutrient-deprivation and ROS generation. This leads to the transcriptional and post-transcriptional upregulation of the BH3-only, pro-apoptotic members of the BCL-2 family proteins (BIM, BID, PUMA, NOXA, BMF, BAD, BIK and HRK) [48,49]. Increased expression of these pro-apoptotic proteins overcomes the anti-apoptotic threshold set by pro-survival members of the BCL-2 family (BCL-2, BCL-XL, BCL-W, MCL-1 and A1/BFL1) in order to activate the mitochondrial pore forming proteins BAX and BAK [49,50]. BAX and BAK (and also BOK) are multi-BH domain containing members of the BCL-2 family which, upon oligomerisation, are responsible for mitochondrial outer membrane permeabilization (MOMP) [51]. MOMP is often deemed the ‘point of no return’ in terms of cell death activation as the subsequent apoptosome-mediated (cytochrome c/Apaf-1/Caspase-9) activation of the executioner caspases-3 and -7 results in the widespread destruction of the cell and its contents [52,53]. In contrast, extrinsic apoptosis is mediated by extracellular signalling molecules which act as ligands for membrane spanning members of the tumour necrosis factor receptor (TNFR) super family known as death receptors [54]. Upon ligand-induced receptor clustering, death receptors such as TNFR1, TRAIL-R1/DR4, TRAIL-R2/DR5 and CD95/Fas recruit intracellular adaptor molecules (such as FADD and TRADD) through death domain (DD) specific interactions which ultimately leads to the formation of death inducing signalling complexes (DISCs) [55,56,57]. Such complexes contain the initiator procaspases, procaspase-8 and procaspase-10 [58,59,60], which bind to the adapter molecules via homotypic death effector domain (DED) interactions in order to become catalytically active [61]. Regulation of procaspase-8 activation at the DISC is determined by its critical regulator, FLIP (*CFLAR*) [48]. Three isoforms of FLIP are found in humans—FLIP long (FLIP_L_), FLIP short (FLIP_S_) and FLIP raji (FLIP_R_) [62,63,64]. Due to their truncated c-terminus, FLIP_S_ and FLIP_R_ are bona fide inhibitors of procaspase activity. In contrast, FLIP_L_ has been described as a pseudo-caspase which can either promote or inhibit procaspase-8 activation based on its relative recruitment to the DISC [65]. In so called ‘type I’ cells activation of caspase-8 at DISCs can directly lead to the induction of cell death through direct effector caspase-3 and -7 activation, however in ‘type II’ cells, signal amplification via the mitochondria is necessary in order to fully activate apoptosis [66]. This involves crosstalk between both the extrinsic and intrinsic apoptotic pathways which is facilitated by caspase-8 and caspase-10 mediated proteolytic cleavage of the BH3-only protein, BID, leading to MOMP and ultimately cell death [67].

Given that apoptosis is driven by both intra- and extra-cellular stress signals, p53 is able to transcriptionally upregulate the expression of target genes and proteins involved in both the extrinsic and intrinsic apoptotic signalling pathways (Figure 2) [68,69]. Key components of the extrinsic apoptotic pathway, the death receptors TRAIL-R2/DR5 and Fas, are well-established direct p53 transcriptional targets upregulated in response to various p53 activatory stress stimuli [70,71]. However, the physiological importance of such death-receptor upregulation is somewhat controversial in terms of their direct cell-death inducing properties. Typically, p53 activation resulting from DNA-damage inducing agents (such as UV-radiation or chemotherapy) does not require extrinsic/death-receptor pathway activation in order to induce cell death [72]. The upregulation of Fas and TRAIL-R2/DR5 may, however, serve to sensitise cells to killing by proximal or recruited death-receptor-ligand-expressing immune cells. To this end, studies have demonstrated that chemotherapy-induced, p53-mediated TRAIL-R2/DR5 upregulation sensitises cells to recombinant ligand binding which facilitates the induction of cell death [73,74]. Thus, p53-mediated death receptor upregulation may enhance the response to cytotoxic damage by priming the cells for elimination by TRAIL or FasL expressing immune effector cells. Interestingly, our recent work identified FLIP as a direct p53 target whose expression is increased as an early response to p53 stabilisation [75]. Sufficiently high levels of the long isoform of FLIP, FLIP_L_ abrogates the function of caspase-8, rendering cells insensitive to extrinsically derived stimuli [65,76]. This seemingly paradoxical role of p53 in transcriptionally regulating the expression of both pro- and anti-apoptotic members of the extrinsic apoptotic pathway may have important roles in preventing cell death *en masse* whilst simultaneously priming cells for death in response to specific stimuli. Indeed, knock-down of FLIP_L_ sensitises cells to p53 induced apoptosis. In the first instance this is driven in a TRAIL-R2/DR5 dependent manner, however, subsequent p53-mediated PUMA upregulation in the absence of FLIP_L_ facilitates the activation of intrinsic apoptosis [75].

Indeed, p53 plays a pivotal role in regulating the transactivation of multiple targets involved in the activation of the intrinsic apoptotic pathway [68]. This includes the BH3-only proteins PUMA and NOXA, as well as the mitochondrial pore forming protein, BAX [77,78,79,80]. Additionally, in response to DNA damage, p53 also transcriptionally upregulates the expression of the apoptosome adapter, Apaf-1, which facilitates executioner caspase activation following MOMP [81,82]. However, the relative contribution of these canonical p53 targets to the induction of p53-dependent apoptosis appears to be quite complex. For example, in the presence of p53 overexpression or in response to p53-activating, DNA-damage-inducing agents loss of PUMA expression abolishes p53-dependent apoptosis, indicating that PUMA is an essential mediator of the p53-dependent apoptotic response [83,84]. However, simultaneous loss of both PUMA and NOXA in response to γ-radiation or drug-induced p53 activation confers more resistance to apoptosis than loss of PUMA alone [77,85]. Conversely, NOXA has been reported as the more essential mediator of UV-radiation-induced apoptosis [86], suggesting that a certain degree of redundancy may exist whereby the critical mediators of the p53 response may vary based on cell type and stimuli. Moreover, whilst BAX and Apaf-1 unquestionably regulate p53-dependent apoptosis, the critical nature of these proteins in the overall apoptotic response means that other measures are in place to regulate their expression in the absence of p53. This has been demonstrated as mice thymocytes lacking p53 display normal levels of BAX and Apaf-1 and undergo apoptosis to a similar degree [87]. Therefore, the p53-induced expression of these targets may serve to enhance the sensitivity of the p53-dependent apoptotic response rather than essentially contributing to this process [69].

Interestingly, a non-transcriptional role for p53 in the regulation of intrinsic apoptosis has also been proposed wherein p53 can directly bind to and activate BAX at the mitochondrial membrane to induce MOMP and cell death [88]. Additionally, studies have suggested that following genotoxic activation p53 can translocate to the cytoplasm and sequester anti-apoptotic members of the BCL-2 family such as BCL2, BCL-XL, and MCL-1 in order to liberate pro-apoptotic BAX, BAK, and BIM repression [89,90]. Thus, the transcriptional-dependent and -independent roles of p53 in the intrinsic apoptotic pathway likely operate in concert to ensure the prompt and efficient induction of cell death. Importantly, the role of p53 in cell death has recently been shown to extend beyond the conventional induction of apoptosis. Novel functions in non-canonical cell death pathways—such as necroptosis, ferroptosis, and pyroptosis—are beginning to emerge which highlight alternative, potentially targetable mechanisms for p53-induced tumour suppression [91].

In contrast to the relatively immune inert, non-inflammatory processes of apoptosis, necrosis represents an immune stimulatory, inflammatory form of what was originally considered ‘accidental’ cell death induced by extreme perturbations of the cellular milieu. However, regulated activation of this pathway is now known to occur in the form necroptosis [2]. Activated in response to TNF and FasL, necroptosis is dependent on the function of two related kinases, receptor-interacting protein kinase-1 (RIPK1) and -3 (RIPK3) [92]. Accumulation of an intracellular complex containing RIPK1, FADD, and procaspase-8 results in the recruitment and subsequent auto-phosphorylation of RIPK3. Phosphorylated RIPK3 activates MLKL (mixed lineage kinase domain pseudo-kinase) which oligomerises to form pores in the cell membrane, disrupting ion flux and resulting in inflammation and immunogenic cell death [92,93]. Importantly, low levels of cellular IAPs (inhibitor of apoptosis proteins) and caspase-8 inactivation are required to propagate necroptotic signalling [94]. As previously mentioned, p53-activation induces the expression of FLIP_L_ which when present at sufficient levels can block caspase-8 activation and thus may contribute to the regulation of necroptosis [75]. Additionally, in response to DNA-damage and ROS generation p53 reportedly transactivates cathepsin Q and directly interacts with cyclophilin D (cypD); both of which contribute to necroptotic cell death [95,96]. Indirect regulation of RIPK1 and RIPK3 via the p53 induced necrosis-related factor (NRF)-miR873 interaction has also been recently identified [97]. Thus, the role of p53 in necroptosis continues to be explored as the immunogenic nature of necroptotic cell death may be beneficial to enhance immune-mediated clearance of tumour cells.

Ferroptosis is described as a regulated form of cell death which is dependent on intracellular microenvironment perturbations such as iron availability and ROS generation and is characterised by the accumulation of lipid peroxides [2]. Various studies have demonstrated that p53 can regulate the induction of ferroptosis through different mechanisms and that is likely an important component of p53′s tumour suppressive function [98,99]. Specifically, p53-mediated transactivation of SAT1 or GLS2 expression or inhibition of SLC7A11 expression result in increased levels of lipid peroxides and ROS required for ferroptotic cell death, whilst p53-mediated activation of p21 or inhibition of DPP4 suppresses ferroptosis.

Pyroptosis is an inflammatory form of regulated cell death morphologically characterised by cell swelling and membrane perforation and molecularly by inflammosome-induced caspase-1 mediated gasdermin-D (GSDMD) cleavage or caspase-3 mediated gasdermin-E (GSDME) cleavage [2,100]. Activation of NOD-like receptors (NLRP1, NLRP3, NLRC4) initiates pyroptotic signalling in response to bacterial infection and other stresses, in order to facilitate pathogen clearance and enhance the innate immune response [100]. In a model of lung cancer, p53 was reported to directly bind to and activate NLRP3 thus promoting pyroptosis [101]. Moreover, p53 has also been reported to transactivate caspase-1 and members of the gasdermin family [102,103]. However, the precise functional role of p53 in pyroptosis remains to be fully determined.

In addition, p53 has an extensive role in regulating cellular metabolism and autophagy (reviewed in [104,105]). Autophagy is an indispensable mechanism through which non-transformed cells maintain metabolic homeostasis by implementing protein quality control and recycling molecular machinery [106]. A complex interplay exists between autophagy and p53 whereby autophagy suppresses p53 and p53 activates autophagy [105]. The context dependent nature of this relationship has major implications for the resulting cell fate as autophagy typically promotes cell survival rather than cell death. However, in particular genetic and environmental contexts autophagy can also result in cell death [107]. Truly autophagy-dependent cell death is molecularly distinct from apoptosis and directly involves components of the autophagic machinery [2]. Nonetheless, considerable overlap also exists whereby the activation of autophagy leads to the regulation of apoptotic cell death in which p53 is thought to play an instrumental role [108]. Nuclear p53 transcriptionally upregulates a number of targets involved in autophagy such as *DRAM1* and *ULK1* [109,110]. DNA damage-regulated autophagy modulator (DRAM), upregulated by stress-activated p53, is a lysosomal protein with prominent roles in the autophagic response and promoting apoptosis [109,111]. Moreover, the p53 transcriptional targets PUMA and BAX have also been implicated in the induction of autophagy which contributes to apoptosis [112]. Conversely, cytoplasmic p53 reportedly facilitates the degradation of Beclin1/*BECN1*, a critical mediator of autophagy, thus p53 can also repress this process [113]. Whether p53-induced autophagy contributes to tumour suppression or survival appears to be entirely context dependent, as inhibiting autophagy can both promote and suppress the p53-mediated apoptotic response [108,111,114].

## 4. Immunogenicity of Cell Death

Emerging evidence has revealed that p53 also plays an important role in immunity, that is mediated at least in part, by its ability to induce cell death (Figure 2) (reviewed in [115,116]). Many viruses, including those associated with tumour development, activate a number of different cellular stress signals that stimulate a cell-death-inducing p53 response. Thwarting this ‘innate’ immune response is essential for the survival of the virus, thus just as cancer cells must circumvent p53 activation, so too must the infected cells. Indeed, many of the discoveries elucidating the anti-cancer functions of p53 have stemmed from research into the capacity for tumorigenic viruses to inactivate p53 [115]. Burgeoning literature support the critical role of p53 in modulating immunity and inflammation and have been extensively reviewed [117,118]. For example, p53 has been shown to have important functions in modulating the interferon response, cytokine production, TLR function and in immune checkpoint regulation. Key targets involved in such pathways include interferon (IFN) regulatory factor 5 (IRF5), IRF9, IFN-stimulated gene 15 (ISG15), toll-like receptor 3 (TLR3), and CC chemokine ligand 2 (CCL2), all of which contribute to the p53-mediated immune response [119,120,121,122,123].

Therefore, in order to escape p53-mediated immune detection and to avoid triggering p53-induced cell death, many tumour-associated and non-tumour associated pathogens have developed efficient yet often quite complex mechanisms for regulating p53 activity. The human papilloma virus (HPV), for example, utilises the viral E6 and E7 proteins to abuse host machinery and dysregulate p53 activity by enhancing p53 protein degradation and disrupting its association with the DREAM complex, respectively [124,125]. Ultimately, in cases of high-risk HPV infection (which accounts for some 70% of cervical cancers) this leads to aberrant cell-cycle regulation and malignant transformation. Similarly, the gastric cancer associated bacterium, *Helicobacter pylori* (*H. pylori*) dampens the p53 response in order to propagate infection [126]. Equipped with oncogenic CagA (cytotoxin-associated gene A), *H. pylori* can promote degradation of p53 through complex CagA-p53 related mechanisms (Figure 2). This includes binding of CagA to the p53 activatory protein ASPP2 (apoptosis-stimulating protein of p53), resulting in the inhibition of p53-mediated transcription and the induction of its proteasomal degradation [127]. Enhanced proteasomal degradation of p53 is also mediated by increased phosphorylation of Akt and MDM2 in *H. pylori* infected cells [128]. In contrast, certain pathogens are known to require functional p53 or even to disparately regulate p53 activity throughout their lifecycle in order to survive. This strategy is observed for viruses such as *HIV-1* (human immunodeficiency virus) in which attenuation of p53 dampens the initial anti-viral response whilst subsequent activation of p53 facilitates the dissemination of the viral infection [129,130]. This complicated interplay between p53 and the microbiome is further exemplified by the recent finding that, in particular regions of the gut, bacteria-released gallic acid can actively switch mutant p53 function from tumour-suppressive to oncogenic by interfering with its ability to abrogate Wnt signalling [131]. Dysregulation of the myriad mechanisms that normally control p53 activity and stability have been described in response to both viral and bacterial infection and are reviewed elsewhere [132,133].

However, upon sufficient microbial- or indeed oncogenic-stress detection, the immune-mediated cell death, and tumour-suppressive functions of p53 become activated. Both cell-autonomous and non-cell autonomous mechanisms have been described which modulate the immune response following p53 activation. For example, in human cancer cells, p53 has been shown to upregulate the transcriptional expression of the natural killer (NK) cell ligands UL16-binding protein 1 (ULBP1) and ULBP2 thus facilitating NK cell mediated tumour clearance [134,135]. Studies have also demonstrated a role for p53 in the regulation of major histocompatibility complex class I (MHC1) cell surface expression which is crucial for appropriate cytotoxic T-cell function. Endoplasmic reticulum aminopeptidase 1 (ERAP1) and transporter associated with antigen processing 1 (TAP1) are responsible for antigen precursor trimming and loading onto MHC1 proteins are transcriptionally regulated by p53 (Figure 2) [136,137]. Defects in ERAP1 have been linked to reduced MHC1 expression and impaired T-cell function [138], indicating that p53 contributes to the effective functioning of this pivotal, cell autonomous mechanism of immune surveillance and T-cell killing. Moreover, in a model of lung cancer, infection with the *H1N1* influenza virus resulted in p53 activation, ERAP1 upregulation and an increase in MHC1 expression highlighting the innate role of p53 as a viral defence mechanism [137].

Notably, non-cell autonomous mechanisms of p53-mediated immune regulation and tumour suppression have also been described. In addition to cell intrinsic functions such as the activation of cell-cycle arrest and apoptosis, p53 can also activate senescence and by extension the senescence associated secretory phenotype (SASP) [139]. Activation of the SASP involves the production and secretion of immunomodulatory factors and inflammatory cytokines as well as components which modulate the extracellular matrix (ECM) which together regulates the tumour microenvironment. This has important implications for tumour growth and development with studies highlighting both tumour-suppressive and tumour-permissive outcomes in response to SASP activation [139]. Importantly, in a fibrosis-associated liver cancer model, the p53-induced SASP has been shown to modulate macrophage polarisation in favour of the M1-state thus facilitating the removal of senescent cells and promoting an anti-tumour immune response (Figure 2) [140]. As has previously been alluded to the p53-dependent regulation of such immuno-modulatory factors—e.g., IFNγ and IL-6—and indeed the activation and regulation of the SASP involves co-operativity with the inherently immuno-regulatory NFκB pathway.

Whilst the p53-dependent regulation of molecules involved in inflammation and immunity is important in terms of tumour-suppression, recent evidence also suggests that the p53 may play a critical role in the regulation of autoimmunity [141]. Indeed, autoantibodies to p53 have been detected in patients with systemic lupus erythematosus (SLE) and other autoimmune diseases [142]. p53 deficiency has also been closely linked to autoimmune and inflammatory conditions in mice suggesting that wild-type p53 normally functions to prevent the inappropriate activation of an immune-mediated cell death response [143,144]. Mechanistically, the p53-dependent transactivation of forkhead box P3 (*Foxp3*) has been shown to contribute to the induction of T_reg_ cells in mice which may be critical in suppressing autoimmunity [144].

Cell death resulting from p53 activation typically induces tolerogenic cell death which is best exemplified by its prominent role in apoptosis [145]. Tightly packaged, so called ‘apoptotic bodies’ containing cellular debris are rapidly engulfed and discarded by neighbouring cells and macrophages. This prevents exposure of immune-stimulatory and inflammatory molecules to the surrounding tissue which could otherwise result in the development of auto-immunity and disease [145]. Importantly, it has recently been reported that p53 actively regulates not only the induction of apoptosis but also the post-apoptotic clean up. Yoon et al. demonstrated that p53 regulates the expression of death domain 1 alpha (DD1α/*VSIR*), a transmembrane protein of the immunoglobin family [146]. In response to radiation, the p53-dependent transcriptional upregulation of DD1α resulted in increased protein expression and promoted the clearance of apoptotic cells by phagocytes (Figure 2). DD1α expression on the surface of T-cells was also shown to result in immunosuppression and DD1α-deficient mice were reported to spontaneously develop autoimmunity [146]. This represents a critical node through which p53 can regulate cell-fate in a non-cell-autonomous manner, i.e., by regulating the cell-death-induced immune response in order to preserve tissue function. Whilst in normal circumstances this process is critical in preventing organ damage and maintaining homeostasis, in cancer cells this may paradoxically lead to tumour development.

Indeed, p53 has also been shown to regulate the expression of immune-checkpoint components such as programmed death 1 (PD1/*PDCD1*) and programmed death ligand 1 (PD-L1/*CD274*) [146]. Although such studies propose that p53-mediated upregulation of PD1/PDL-1 may constitute another means of immune escape exploited by cancer cells retaining wild-type p53 expression (Figure 2), this may be context dependent as recent observations suggest that p53 can also reduce anti-immunogenic PDL-1 expression [147]. This has been shown to be executed by microRNA-34a (mir34a), a well-established p53 transcriptional target and key regulator of p53-mediated tumour suppression. Mir34a directly binds to the 3′ untranslated region of the gene encoding PDL-1, preventing its transcriptional expression and antagonising T-cell exhaustion [147]. In contrast, higher PDL-1 expression in p53 mutant/deficient tumours with reduced mir34a expression may lower immunogenicity and facilitate the survival of immune-cold and potentially more aggressive cancers.

Additionally, the p53/mir34a axis has been further implicated in preventing CD8+ T cell exhaustion by reducing the secretion of Golgi reassembly and stacking protein 55 kDa (G55)-dependent protein [148]. G55 is a Golgi stacking protein which contributes to secretory vesicle biogenesis, the contents of which have been shown to enhance proliferation and invasion in p53-deficient lung cancer cells by modulating the tumour microenvironment [148]. Thus, the p53-dependent, mir34a-mediated silencing of G55 expression constitutes an important mechanism through which the emerging functions of p53 in Golgi regulation can modulate the tumour response. Moreover, mutant p53 has recently been shown to induce Golgi tubulo-vesiculation driving a pro-metastatic secretome which modulates the tumour microenvironment in order to enhance tumour growth and dissemination [149].

p53 has also been shown to play a role in the release of extracellular vesicles, and in particular exosomes, is gaining significant interest in cancer research and immunology owing to the increasing importance of the complex interplay between cancer cells and the tumour microenvironment [150,151]. This includes effects on adjacent cancer cells, resident immune cells as well as cancer associated fibroblasts (CAFs) and the ECM which together determine cancer initiation, progression, and response to treatment. In response to stress, p53 has been shown to transcriptionally regulate TSAP6, a multi-pass transmembrane protein that facilitates exosome secretion [150]. Exosome size and cargo are also modulated by p53 activity with multiple anti-tumorigenic effects described. This includes regulation of exosome dependent immune surveillance as well as repression of exosomal *TP53* miRNAs destined to inactivate p53 in CAFs [151,152].

Given the emerging complexity highlighted above, the relationship between p53, immunity and cell death likely has many more secrets to reveal, since for example recent data demonstrates a crucial role for the extrinsic, p53-regulated, cell death DR5/Caspase-8/FLIP axis in amplifying cell death activated by CAR-T cells [153]. This indicates that the balance of p53 activity in cancer cells may additionally determine how cancer cells respond to T-cell responses that are critical for immunotherapies. Thus, determining the way in which cells die in response to p53 activation is of critical importance from a cancer therapeutics perspective with much still to be uncovered in the promising field of immuno-oncology.

## 5. (De)Regulation of p53-Induced Cell Death

In unstressed, non-transformed cells p53 protein levels are maintained at low, almost undetectable levels in order to avoid aberrant activation of cell-cycle arrest and cell death. A complex regulatory network therefore exists upstream of p53 which under basal conditions ensures homeostasis and in response to stress stimuli rapidly facilitates p53 stabilisation and activation. The activity of such regulatory networks, and subsequently the p53 protein itself, is governed by an extensive array of post-translational modification (PTM) events which have been shown to directly affect p53-induced target activation and ultimately cell fate [39]. Not surprisingly, cancer cells have developed multiple strategies to exploit and dysregulate key components of the p53 signalling network in order to mitigate its tumour suppressive functions and evade cell death [154,155]. The simplest way to inactivate p53 is to mutate and/or lose the wild-type copy of the gene, yet while this happens in more than fifty percent of all cancers, why cancers from certain tissues or indeed molecularly defined sub-types selectively evolve to maintain wild-type p53 and suppress activity through altering upstream activator/repressors or downstream effectors to affect cell fate outcomes (e.g., arrest vs. death) remains poorly understood.

**Figure 3:**
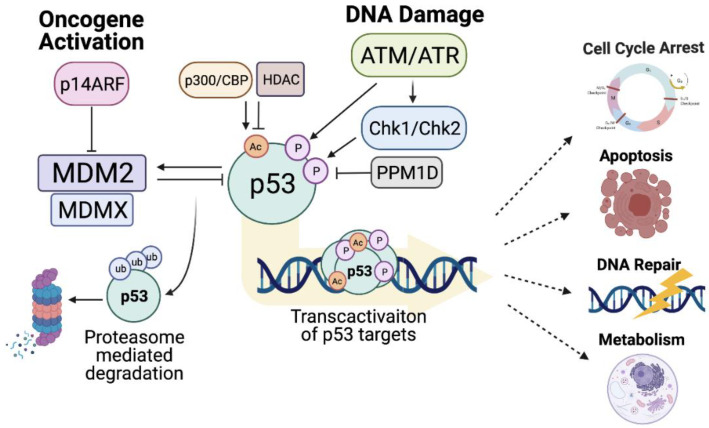
Regulation of p53 stability and transcriptional activity. Schematic representation of the key proteins involved in the regulation of p53 activity and stability and the core downstream pathways regulated in response to p53 activation. Created with BioRender.com (accessed on 15 May 2021).

The best described and dominant regulator of p53 activation is the E3-ubiquitin ligase, mouse double minute 2 (MDM2) (Figure 3) [156,157], which binds to and inhibits the N-terminal transactivation domain of p53. Through its E3-ligase activity MDM2 also targets p53 for proteasomal degradation via poly-ubiquitination of multiple lysines within the p53 CTD, thereby reducing p53 protein stability [158,159]. MDM2 binding to p53 can also suppress the transcription factor activity of p53 through competitive binding and inhibition of activatory co-factors and by facilitating p53 nuclear export [160,161]. Importantly, *MDM2* gene expression is itself under the transcriptional control of p53, thus an auto-regulatory loop exists wherein MDM2-mediated p53 inhibition concomitantly reduces MDM2 expression [157].

Canonically, MDM2 functions as homodimer, although heterodimers of MDM2 and MDMX (*MDM4*) also exert dominant negative effects on p53 activity [162]. MDMX is a homologue of MDM2 which, due to its inability to form a homo-dimeric structure, does not possess inherent E3-ligase activity. Nonetheless, MDMX can also negatively regulate p53 activity through MDM2-dependent and independent mechanisms (Figure 3) [163]. Knockout studies in mice have demonstrated that loss of either protein results in embryonic lethality [164,165], highlighting the non-redundant nature of their roles. Moreover, simultaneous loss of p53 has been shown to rescue this phenotype [164,165], thus establishing the MDM2/MDMX-p53 axis as an essential regulatory mechanism which controls cell fate during development.

This prominent role of MDM2-mediated p53 inhibition in facilitating cell survival is frequently exploited by cancer cells in order to suppress the anti-tumour activity p53 [166]. Amplification of *MDM2* has been detected in multiple tumours including sarcomas [167], lung cancer [168] and colorectal cancer [169], moreover, MDM2 over-expression is also linked to poor prognosis and increased chemotherapeutic resistance [170]. Furthermore, a single nucleotide polymorphism (SNP) in the *MDM2* promoter has been shown to create a binding site for the transcription factor SP1 which increases MDM2 expression, abrogates p53 activity and accelerates tumour development [171]. The MDM2-p53 axis plays a sentinel role in sensing homeostatic and oncogenic stress. For example, ribosomal/nucleolar stress (a rheostat for cellular homeostasis) leads to release of nucleolar RPL5/11 which can inhibit MDM2 interaction and stabilise p53 [172]. MDM2 activity can conversely be activated by phosphorylation upon activation of proto-oncogenes such as Akt [173].

Additionally, the MDM2-p53 axis is harnessed by the tumour suppressor p14^ARF^ (a nucleolar protein encoded by an Alternative Reading Frame of the *INK4a*/*CDKN2A* locus) as a key sensor of hyperproliferative stimuli (Figure 3) [174,175]. Aberrant mitogenic signalling and oncogenic stress lead to increased ARF expression, which results in MDM2 being sequestered in the nucleolus. In addition to MDM2 inhibition, ARF also negatively regulates the activity of ARF-BP1 (ARF binding protein 1) which also directly binds and ubiquitinates p53 [176]. p14^ARF^ stabilisation and activation of p53 typically culminates in the induction of cell-cycle arrest, however, is response to adequate stimuli cell death can also ensue. In vivo analyses have demonstrated that ARF deficient mice are highly tumour prone, thus this pathway represents a primary mechanism of tumour surveillance [177]. Inactivating mutation or deletion of p14^ARF^ is frequently reported in human cancers alongside non-mutational mechanisms such as increased gene silencing by methylation, expression of splice variants and negative regulation by the oncogenic transcription factor Twist1 (reviewed in [178]). Whilst loss of p14^ARF^ function may promote oncogenesis via p53 dysregulation, overexpression of p14^ARF^ has also been correlated with mutant p53 expression and thus a more aggressive phenotype [179]. Utilising p14^ARF^ expression as a prognostic biomarker in cancer therapy has therefore been somewhat controversial [178].

Key regulators of the DNA-damage response (DDR) pathway, ataxia-telangiectasia-mutated (ATM) and ataxia telangiectasia and Rad3-related (ATR) are critical for maintaining genomic integrity and have a key role in regulating p53 activation through PTM [180,181]. DDR activated by double or single strand breaks is driven by ATM and ATR respectively, which subsequently trigger phosphorylation of downstream kinases, checkpoint kinase 1 and 2 (Chk1/*CHEK1*, Chk2/*CHEK2*) (Figure 3). Phosphorylation of p53 by Chk1/Chk2 [182], or directly by ATM [180], occurs at the N-terminal transactivation domain and blocks the negative interaction of MDM2. Multiple residues within this region (S15, S20, S46, and T18) undergo phosphorylation which can also be induced by other stress-induced kinases such as HIPK2 (homeodomain-interacting protein kinase 2) [183]. While phosphorylation of S15 is often described as a nucleation event which promotes further p53 modification, particular phosphorylation events such as S46 phosphorylation have been shown to regulate p53 activity in favour of cell death by transcriptionally activating the pro-apoptotic mitochondrial associated gene *TP53AIP1*/p53AIP1 (p53-regulated Apoptosis-Inducing Protein 1) [183,184]. Importantly, phosphorylation-induced p53 activation can be repressed by *PPM1D*/WIP1 which actively dephosphorylates the p53 N-terminus thus adding an additional degree of complexity to the regulation of p53 and its functional output (Figure 3) [185]. Nevertheless, in response to adequate stimuli/DNA damage, p53 N-terminal phosphorylation events efficiently disrupt the binding of MDM2 to this region, leading to increased p53 stability and transcription factor activity. Additionally, the phosphorylation-dependent dissociation of MDM2 from p53 facilitates the binding of transcriptional co-activators which play an important role in determining the p53 response and therefore cell fate. For example, phosphorylation of p53 enhances its interactions with co-factors such as ASPPs (Apoptosis Stimulation of p53 protein), which induce p53 activity on the promoter of pro-apoptotic target genes [46]. Phosphorylation events at the TAD can also facilitate the recruitment of the acetyltransferase p300 that promotes acetylation and thus activation of p53 transcription activity [186].

In addition to phosphorylation-specific regulation, p53 undergoes extensive acetylation mediated by acetyltransferases such as p300, CBP (CREB-binding protein) and PCAF (p300/CBP-associated factor complex) (Figure 3) (reviewed in [187]). Moreover, acetylation is indispensable for p53 activation [188]. p53 is rapidly acetylated in response DNA damage and particular acetylation events have been shown to regulate p53-induced cell fate by transcriptionally favouring the expression of genes involved in either cell-cycle arrest or apoptosis [187]. The lysine rich CTD of p53 is highly decorated with acetylation marks (most frequently at K370, K372, K373, K381, K382, and K386) although acetylation also occurs within the DNA binding domain (K120 and K164) and the oligomerisation domain (K320) [39]. CTD acetylation stabilises p53 protein expression and enhances its transcriptional activity by competitively inhibiting MDM2-mediated ubiquitylation at the same lysine residues [189]. Additionally, CTD acetylation can also inhibit protein–protein interactions which in unstressed/unacetylated cells act to suppress p53 transcriptional activity. For example, under homeostatic conditions, the positively charged, lysine-rich, p53 CTD acts as a docking site for acidic domain containing proteins such as the proto-oncogene, SET, leading to the formation of a p53-SET transcriptionally repressive complex [190]. However, stress-induced acetylation of p53 lysine residues neutralises their positive charge, abolishing the CTD interaction with SET and promoting p53 activation [190]. Interestingly, whilst p53 acetylation at eight key lysine residues within the CTD is said to be indispensable for its overall tumour suppressive function, particular acetylation events have also been shown to regulate the p53 response in favour of either cell-cycle arrest or cell death. Studies have demonstrated an important role for K320 acetylation in the regulation of cell-cycle arrest [191], whereas acetylation of lysine K120 by the acetyltransferase Tip60 is crucial for p53-dependent apoptosis and dispensable for cell-cycle arrest [192,193]. Importantly, multiple de-acetylases (histone deacetylases 1, 2, and 3 (HDAC1/2/3) and SIRT1) actively counteract the acetyltransferase activity of p53 co-activators and can therefore inhibit p53 transcriptional activity and tumour suppressive functions [194,195].

Importantly, cancer cells frequently dysregulate the processes which govern p53 post-translational modification in order to attenuate its tumour suppressive functions [155]. This can occur through loss of function mutations or deletions in ATM/ATR, or their downstream pathway components, which perturbs p53 phosphorylation and activation in response to DNA damage and significantly increases the likelihood of tumour progression [196]. Moreover, given the importance of acetylation in regulating the expression and activity of p53, as well as facilitating the formation of euchromatin and enabling transcription factor binding to underlying DNA targets, it is perhaps not surprising that cancer cells frequently overexpress HDACs as a means of attenuating tumour suppressive transcriptional functions of wild-type p53 [197] (Figure 3). Overexpression of the Class I-specific nuclear HDACs (HDAC1, HDAC2, and HDAC3) has been associated with poor prognosis in multiple myeloma [198] and in colorectal cancer where Class-I HDAC expression has been described as an independent prognostic factor [199]. Moreover, high expression of HDAC1 has also been shown to correlate with gastrointestinal tumour progression and poor prognosis [200]. HDAC1 is notably recruited by MDM2 in order to deacetylate and destabilise p53 expression resulting in a diminished transcriptional and tumour suppressive response [201]. Similarly, HDAC2 has been shown to regulate p53 activity in response to ionising radiation wherein p53-dependent survivin downregulation, mediated by HDAC2-downregulation-induced MDM2 destabilisation, enhanced the apoptotic response [202].

## 6. Targeting p53 to Induce Cell Death

Developing therapeutic strategies aimed at reviving the latent activity of wild-type p53 in cancer cells represents an extremely valuable clinical opportunity to exploit the innate tumour suppressive function of this fundamental cellular protein. Indeed, restoration of p53 in various in vivo cancer models results in tumour regression highlighting the inherent potency of this tumour suppressor and its profound role in abrogating the survival of cancer cells [203,204,205]. Moreover, a more robust response to standard-of-care treatments such as cytotoxic chemotherapy and ionising radiation is often observed in the presence of functionally active p53. Thus, enhancing the expression and tumour suppressive activity of wild-type p53 has the potential to significantly alter cancer progression and improve responses to current treatments. However, as highlighted throughout this review, sub-lethal activation of p53 may paradoxically promote tumour progression by activating anti-apoptotic proteins, autophagy, alternative cell death pathways as well as cellular senescence (reviewed in [206]) with tumour promoting properties that may in turn confer resistance to p53 activating therapies such as chemotherapy. This may explain why some tumours retain wild-type p53 expression, i.e., to exploit its pro-survival functions. Carefully considered combination treatments targeting such mechanisms may therefore be more effective in the treatment of cancer.

Understanding p53 dynamics, particularly at the single cell level, will be critical to this process. A growing body of research now demonstrates that differential cell fates elicited in response to p53 activation are determined by both the degree and duration of the p53 stimulatory signal and by the relative mRNA and protein stability of its transcriptional targets. For example, treatment with ionizing radiation has been shown to induce pulses of p53 protein expression and activity which facilitates the induction of DNA-repair mechanisms and cell survival. In contrast, when p53 expression is sustained over longer periods of time (e.g., following treatment with Nutlin-3A) this can lead to the irreversible activation of senescence or indeed cell death [207]. Moreover, recent analysis has demonstrated that a subset of cells can switch from oscillating to sustained p53 dynamics several days after undergoing irradiation. This results from cell-cycle arrest escape in the presence of DNA-damage which subsequently stabilises p53 expression [208]. Thus, the amplitude, frequency, and duration of the p53 response can significantly alter the cellular outcome.

Subsequently, a threshold mechanism of cell fate regulation has been described whereby the relative level and duration of p53 expression, and those of its transcriptional targets, work in concert under certain cellular conditions and contexts to induce cell-cycle arrest, apoptosis and other cell fates. Significant/sustained levels of p53 activation are therefore required in order to overcome the “apoptotic threshold” and induce cell death, whereas below this threshold cells typically undergo cell-cycle arrest [209]. Interestingly, a recent study by Paek et al. has also demonstrated that although a threshold for p53 activity must be reached in order to activate cell killing, this threshold significantly increases over time and thus p53-induced cell fate is also regulated in a dynamic temporal manner [210]. This process has been attributed to the treatment-induced upregulation of negative regulators of cell death which are concomitantly induced alongside pro-apoptotic p53 targets in response to chemotherapy [210]. Over time, the accumulation of such anti-apoptotic proteins increases the threshold of p53 activation that is required to elicit a cell death response and may even contribute to the emergence of resistance in response to chemotherapeutic agents which result in fractional cell killing.

Indeed, distinct patterns of gene expression have recently been described in single-cell transcriptome analysis of CRC cells wherein fractional cell killing was observed. Heterogenous DNA-damage responses within a single population of colon cancer cells treated with 5-FU resulted in three distinct cell fates, namely apoptosis, cell-cycle checkpoint and stress resistance, each with a corresponding gene expression profile [211]. Such analyses highlight the need to transcriptionally and phenotypically evaluate single cells in order to understand the distinct biology underpinning cell fate within a population in response to chemotherapy. For example, in response to non-lethal doses of chemotherapy, such as during the inevitable decline in drug concentration during treatment, surviving cancer cells have been shown to undergo senescence or re-enter the proliferating population [212,213]. These actively proliferating cells have the potential to influence tumour progression and patient outcome, thus delineating mechanisms which regulate the senescent vs. proliferating cell fate is an area of active research. Interestingly, recent advances in this area have proposed that the cell fate decision may be influenced by the prominent p53-induced target, p21. In an elegant study mapping individual cell responses, Hsu et al. demonstrated that p21 dynamics were critical in determining whether cells were fated to proliferate or senesce after exposure to sub-lethal doses of chemotherapy [214]. Whilst both low and high levels of p21 expression during drug treatment were linked to a senescent cell fate, intermediate p21 levels promoted a proliferative cell fate. This “Goldilocks zone” of p21 expression, which favours cancer cell proliferation, may be induced by current strategies aimed at increasing p53 activity as p21 will also be concomitantly upregulated. The consequences of such a phenomenon require immediate and further exploration and likely have important implications for cancer progression and recurrence.

Advances in our understanding of the mechanisms which canonically regulate p53 stability and function have facilitated the development of numerous small molecules and drugs that exploit vulnerabilities within the p53 regulatory network in tumours retaining wild-type protein expression [14,215]. In p53 wild-type tumours targeting the MDM2-p53 regulatory axis has the potential to reactivate wild-type p53 and inhibit cancer survival [216]. To this end, numerous compounds have been developed which target MDM2 and/or MDMX, p53 and upstream regulators (reviewed in [14]). The first major effort in the development of small molecule inhibitors of MDM2 resulted in the generation of a family of cis-imidazoline analogues termed Nutlins [217]. Nutlin-3A (the lead compound) binds to the hydrophobic pocket of MDM2 which is normally responsible for binding and inhibiting p53 activity. Thus Nutlin-3A stabilises p53 expression and facilitates its transcription factor activity leading to upregulation of putative p53 targets such as the cell-cycle arrest associated cyclin-dependent kinase inhibitor, p21, and the pro-apoptotic BH3-only protein, PUMA [218,219]. Despite regulating both pro-arrest and pro-apoptotic targets simultaneously, Nutlin-3A-mediated MDM2 inhibition typically results in p53-dependent cell-cycle arrest rather than cell death, in all but MDM2-amplified tumours such as liposarcoma [220].

In addition, the ubiquitin specific protease 7 (USP7, HAUSP) has also been the focus of drug development owing to its role in the p53-MDM2 axis [221,222,223]. Inhibiting USP7 (reviewed in [19]) promotes MDM2 destabilisation and degradation [224,225] which liberates the tumour suppressive function of p53. However, UPS7 inhibition can also indirectly regulate p53 target activation; for example, inhibition of USP7 destabilises Tip60 resulting in decreased p53 induced PUMA expression and attenuation of apoptosis [226]. Although the use of small molecule USP7 inhibitors may therefore be context dependent, anti-cancer efficacy has been reported in several pre-clinical models making it an attractive target in cancer therapy.

However, when combined with agents that enhance p53 activity, small molecule inhibition of the MDM2-p53 axis can significantly augment the p53 response in favour of apoptotic cell death. Recent work from our lab has demonstrated that Entinostat (a Class I-specific HDAC inhibitor) can suppress p53-induced FLIP_L_ upregulation in response to treatment with Nutlin-3A and chemotherapeutic agents in models of colorectal cancer [75]. In the first instance, this essentially alleviates the break on caspase-8 activation and results in the induction of TRAIL-R2/FADD/caspase-8-dependent cell death. Interestingly, attenuation of p53-induced FLIP_L_ also appears to regulate the expression of Nutlin-3A-induced p53 transcriptional targets to promote caspase-8-independent cell death at later timepoints. This involves a switch in p53 transactivation which results in the suppression of Nutlin-3A-induced p21 expression and upregulation of Nutlin-3A-induced PUMA expression [75]. Enhanced PUMA expression subsequently mediated p53- and mitochondrial-dependent apoptosis. This work highlights the multi-nodal role of p53 activation in regulating both the extrinsic and intrinsic apoptotic pathway and demonstrates an effective therapeutic angle for the combined treatment of MDM2 inhibitors with HDAC inhibitors.

Unfortunately, clinical translation of the Nutlin-3A analogue RG7112 was hampered by dose-limiting toxicities such as thrombocytopenia and neutropenia which precluded its use in cancer treatment [216,227]. The huge potential in targeting the MDM2-p53 interaction has, however, spurred the development of more potent Nutlin derivatives such as RG7388. This pyrrolidine compound has demonstrated enhanced efficacy at doses which are orders of magnitude lower than those of its predecessors both in vitro and in vivo [228]. As with other MDM2 inhibitors, treatment with RG7388 effectively activates p53, leading to the induction of cell cycle arrest or apoptosis in tumours expressing wild-type p53. Clinical trials involving the use of RG7388 are currently ongoing with hopes for future success. In addition, other classes of small molecule inhibitors have been developed such as the spirooxindoles (e.g., MI773), benzodiazepinediones (e.g., TDP521252), and piperidinones (e.g., AMG232) [229]. Differing in their structural composition and affinity for the key residues in the MDM2 binding pocket, these compounds have also made their way into clinical trials increasingly in combination with targeted therapies [230]. Studies which have reported on the safety and tolerability of such compounds show promise for the use of these agents in the clinical setting, however limited tumour-suppressive single-agent effects has been observed [231]. This highlights the need for improved patient stratification methods mediated by biomarker identification and predictive gene signatures which determine sensitivity to these agents. Moreover, combination treatments which exploit vulnerabilities induced by p53-activation or which facilitate a cell-death driven p53 response represent an alternative clinical strategy for the use of MDM2-inhibitors. Identifying mechanisms of resistance to the induction of cell death following treatment with MDM2-inhibitors will enable specific targeted therapies to be utilised, alleviating blockades in the p53-induced anti-cancer response and harnessing the full potential of these compounds.

As summarised above p53 plays a critical role in regulating the diverse pathways that can influence cell fate decisions between life and death, circumventing p53 activation and cell death is therefore of critical importance in oncogenesis and inevitably modulates response to treatment. Due to the inherent cellular stresses associated with neoplastic transformation, cancer cells may be exquisitely more sensitive to the activation of such cell death pathways when compared to their normal cell counterparts. However paradoxically, sub-optimal activation of p53 and cell death activating pathways in response to treatment has potential to promote survival of at least a subset of tumour cells and potentially influence the ability of the immune system to recognise and kill residual tumour cells. Interestingly, this has been proposed as a means to protect normal cells from the toxicity of DNA damaging agents such as chemo- and radio-therapy by activating non-apoptotic p53 activation using “cyclotherapy” [232,233].

In tumours retaining wild-type p53, targeted agents that stabilise p53 have shown promise but are likely limited clinically by on target toxicities and with such exquisitely targeted agents the spectre of loss through p53 mutation looms large. It is therefore likely that these agents may be most effective when combined with agents that together alter the cell death inducing threshold. For example, MDM2 inhibitors or targeted agents that alter MDM2 activity/stability can augment chemotherapy response [210], be augmented by inhibitors of p53 inactivators such as WIP1 [234], or by inhibitors of IAPs [235], or direct activators of cell death such as BH3 mimetics [236]. Moreover, our work and others have shown that combinations with epigenetic modifying agents including HDACi [75] and bromodomain inhibitors [237] may enhance p53 induced cell death through more than one of these mechanisms and intriguingly, bispecific small molecules targeting MDM2 with BRD4, BCL2, and XIAP present interesting strategies for wild-type p53 containing tumours [238,239,240].

## 7. Conclusions

It is therefore clear that the intensity and duration of p53 activation induced by cancer treatments plays a critical role in determining cancer cell fate and with greater understanding this may be rationally exploited to augment cancer cell death. Much is still to be learned in terms of how the nature of p53-regulated cell death affects interaction with the immune system, the consequence of p53 mutation or loss, and how this can be used to inform novel treatment combinations with agents which regulate cell death and immunity.
